# Uses of X-Ray Diagnosis, or Radiography in Dentistry*Read before the Daviess County Dental Society.

**Published:** 1922-11

**Authors:** J. H. Taylor

**Affiliations:** Owensboro, Ky.


### USES OF X-RAY DIAGNOSIS, OR RADIOGRAPHY IN DENTISTRY*

BY DR. J. H. TAYLOR

The first step should be the apparatus best suited to the work. Any of the specially designed machines made for a dental office which have ease of manipulation and least exposure of operator to rays, and which make the clearest pictures, will in my mind suit the average practitioner. There are two kinds of machines in reference to tubes. That is the gas tube and the Coolidge tube. Not being familiar with the gas tube, I will not be able to criticize or explain its merits, but having used a Coolidge tube I have learned some of its merits, especially when used on a resistance type of machine. There is but one danger with a Coolidge tube, and that is over-exposure, causing the tube to get too hot. Forty to sixty seconds, I believe, is the maximum strength of any Coolidge tube, which is much more than any dental usage would receive.

Where you have to regulate your spark gap on a machine it takes time, and is trying on the patient's nerves. This is because the patient probably never saw an X-ray, and the general appearance of a machine where you have a rotary generator and have to regulate the spark gap, the noise and appearance, seems to frighten them enough to excite their nerves and cause them to move when the exposure begins, resulting in bad pictures with no diagnostic value at all.

In comparison, using a Coolidge tube machine, you are always ready to make an exposure, and after the first exposure the patient feels no ill effects and the operation of getting more than one picture comes easy.

* Read before the Daviess County Dental Society.

It is true that a good deal of this depends upon the assurance of the operator and confidence the patient has in the operator.

As to strength of an X-ray current needed in the dental office for dental radiographs, I believe a 10 M. A. machine will do as good work as a 30 M. A. machine; 10 M. A. is claimed by most operators to give a better picture; that is, a picture of either of the maxillary bones leaves a picture of more diagnostic value than is practicable with 30 M. A. Of course when you use 30 M. A. you cut the time of exposure, but with 10 M. A. your exposure will range from two to four seconds, depending upon the thickness of the cheek.

If the dentist wants a machine to do diagnosis for the M. D's., using the aid of flouroscope and screens, then I would say a 30 M. A. should be used, adapting the same in his dental practice by shortened exposures to correspond with 10 M. A. of regular exposures in making radiographs of the teeth.

The next step I will refer to is the position of patient and machine. With a machine as I mentioned at the first I like the patient to sit upright, looking straight to the front, then we will best get our angle and distance. What we mean by angle is getting the tube on a line that is vertical to the plane that bisects the angle between an imaginary plane of the teeth and the film, so that the length of the image on the film will compare with the real length and size of the teeth. This is absolutely necessary to have a perfect picture. This is especially so in pyorrhea alveolaris, where there is nearly always necrosis and seremunal deposits, and to accurately tell the line where healthy tissue comes. For a full mouth of eleven exposures, we start on upper right third molar and revolve patient as we make the different exposures without touching the machine until the upper left third molar is taken, therewith establishing the angle with the machine, patient retaining same position. Then you start on the inferior left third molar and operate as above described until you have completed the full mouth of pictures. The hardest teeth to X-ray, I find, are the upper molars, so as to get the third or lingual roots to show plain enough to be of diagnostic value. The distance of center of the tube to the patient should be from twelve to eighteen inches—this makes the best radiograph. The way to read a radiograph is by use of a shadow box, so that you can get an even light without a shadow.

Dentists should use the X-ray as confirmation of their different physical diagnoses. Under that head, I will name a list of reasons for use of the X-ray picture or radiograph: To see that you have properly filled your root canals, to locate lost instruments within root canals, to clearly define roots of broken teeth to aid in the extractions of such roots, to show blind apical abscesses and fistulas, to show malerupted or impacted teeth, to show diseased conditions of the anthrum, to show diseases of frontal sinuses, to show fractures of inferior or superior maxillary and to show necrosed bone conditions caused by pyorrhea in both inferior and superior maxillary bones.

I think I have named the most essential features of the use of the X-ray, but another is that if the dentist can back up his diagnosis with X-ray photographs he has good evidence to justify his diagnosis. This is especially so if he lives in a town where he has the use of same, for then there is less excuse for not using the X-ray when results disclose that the radiograph should have been of valuable diagnosis.

I will not describe the developing of dental X-ray films. The first step is to have a dark room, properly equipped so that you can get your films out quickly and to have the assurance of knowing that you did it yourself. At first this seems to be the most dreaded ordeal to deal with, and you will have quite a few failures at first. You can buy your own developer and fixer, or you can secure them from Eastman's. I mean the packages that you buy from the supply house is in the right proportion for the right amount of solution you need for developing dental films. Trays or bowls, such as a medium size soup bowl, are what I use. They are deep enough to hang down without letting films lay on bottom of same, and there is no chance of not getting negative under solution.

I do not believe of running the question of radiographs into the ground, but I do believe that dentists who do not do a good deal of X-ray diagnosis—that is, have pictures made, or make them themselves—do not realize how many diseased mouths and blind abscesses they overlook. It is astonishing to know how many infections of the alveolar process you do not suspect as pyorrhetic, and which by the X-ray show enormous loss of bony tissues. The proper time to treat such conditions is when they first start. It requires an X-ray diagnosis to detect the beginning.

I could say a great deal more about X-ray pictures, but feel that I have laid before you some of the principles of radiography which are of interest.

Owensboro, Ky.

